# Morphology-Dependent Photocatalytic Activity of Nanostructured Titanium Dioxide Coatings with Silver Nanoparticles

**DOI:** 10.3390/ijms25168824

**Published:** 2024-08-13

**Authors:** Nasir Shakeel, Ireneusz Piwoński, Aneta Kisielewska, Maciej Krzywiecki, Damian Batory, Michał Cichomski

**Affiliations:** 1 Department of Materials Technology and Chemistry, Faculty of Chemistry, University of Lodz, Pomorska 163, 90-236 Lodz, Poland; nasir.shakeel@edu.uni.lodz.pl (N.S.); aneta.kisielewska@chemia.uni.lodz.pl (A.K.); michal.cichomski@chemia.uni.lodz.pl (M.C.); 2 Department of Applied Physics, Institute of Physics—CSE, Silesian University of Technology, Konarskiego 22 B, 44-100 Gliwice, Poland; maciej.krzywiecki@polsl.pl; 3 Department of Vehicles and Fundamentals of Machine Design, Faculty of Mechanical Engineering, Lodz University of Technology, Zeromskiego 116, 90-924 Lodz, Poland

**Keywords:** nanomaterials, morphology, titanium dioxide nanorods, photocatalysis, silver nanoparticles

## Abstract

This study aims to improve the photocatalytic properties of titanium dioxide nanorods (TNRs) and other related nanostructures (dense nanorods, needle-like nanorods, nanoballs, and nanoflowers) by modifying them with silver nanoparticles (AgNPs). This preparation is carried out using a two-step method: sol–gel dip-coating deposition combined with hydrothermal crystal growth. Further modification with AgNPs was achieved through the photoreduction of Ag^+^ ions under UV illumination. The investigation explores the impact of different growth factors on the morphological development of TiO_2_ nanostructures by modulating (i) the chemical composition, the water:acid ratio, (ii) the precursor concentration involved in the hydrothermal process, and (iii) the duration of the hydrothermal reaction. Morphological characteristics, including the length, diameter, and nanorod density of the nanostructures, were analyzed using scanning electron microscope (SEM). The chemical states were determined through use of the X-ray photoelectron spectroscopy (XPS) technique, while phase composition and crystalline structure analysis was performed using the Grazing Incidence X-ray Diffraction (GIXRD) method. The results indicate that various nanostructures (dense nanorods, needle-like nanorods, nanoballs, and nanoflowers) can be obtained by modifying these parameters. The photocatalytic efficiency of these nanostructures and Ag-coated nanostructures was assessed by measuring the degradation of the organic dye rhodamine B (RhB) under both ultraviolet (UV) irradiation and visible light. The results clearly show that UV light causes the RhB solution to lose its color, whereas under visible light RhB changes into rhodamine 110, indicating a successful photocatalytic transformation. The nanoball-like structures’ modification with the active metal silver (TNRs 4 Ag) exhibited high photocatalytic efficiency under both ultraviolet (UV) and visible light for different chemical composition parameters. The nanorod structure (TNRs 2 Ag) is more efficient under UV, but under visible-light photocatalyst, the TNRs 6 Ag (dense nanorods) sample is more effective.

## 1. Introduction

Nanoparticle-based photocatalysis shows promise for various applications, including solar energy conversion, water splitting, and environmental pollution treatment [1,2]. Titanium dioxide (TiO_2_) is a highly active and chemically inert photocatalyst that is frequently utilized in both fundamental research and commercial applications. It is preferred due to its non-toxic nature and promising performance. Ongoing advancements in generating and altering TiO_2_ nanoparticles have led to new characteristics and uses in photocatalysis, resulting in enhanced efficiency. The distinctive chemical and physical properties of titanium dioxide can be manipulated by adjusting the nanocrystal structure, size, shape, and organization [3]. Nevertheless, the practical use of TiO_2_ is frequently constrained by its large energy difference between its valence and conduction bands, known as its wide band gap. This limitation confines its ability to convert light into electrical energy to the ultraviolet region of the electromagnetic spectrum, thereby capturing only a small portion of solar energy. In order to address these limitations, significant endeavors have been focused on altering the structure of TiO_2_ nanostructures and integrating metal nanoparticles to improve their photocatalytic efficacy [4]. There are various shapes of TiO_2_ nanostructures, such as nanospheres, nanorods, nanotubes, and nanowires, each with varying levels of photocatalytic efficacy. Nanorods, among other 1D nanostructures, demonstrate superior thermal stability at elevated temperatures due to their crystal structure. Consequently, they exhibit a higher electron transport rate at grain boundaries compared to nanotubes and nanowires [5]. An effective approach involves utilizing TiO_2_ nanorods that are coated with Ag NPs. The distinctive one-dimensional configuration of TiO_2_ nanorods provides a large surface area and facilitates efficient photocatalytic reactions through directed charge transport. The addition of Ag nanoparticles onto TiO_2_ nanorods has two main effects. Firstly, it increases the absorption of light in the visible region by taking advantage of the surface plasmon resonance effect of Ag. Secondly, it enhances charge separation by serving as electron sinks, which leads to a decrease in the recombination rate of photogenerated electron–hole pairs. The process of synthesis of TiO_2_ nanorods hydrothermally has been extensively reported by numerous researchers, who have utilized various substrate materials [6,7,8,9]. Rahmani et al. produced TiO_2_ nanorods (NRs) on silicon and porous silicon (PS) substrates using the hydrothermal method [10]. Chandra et al. developed a hierarchical morphological structure by synthesizing TiO_2_ nanorods that are decorated on vertically aligned silicon nanowire (SiNW) leads. The photocatalytic properties of the SiNW/TiO_2_ nano heterojunction are investigated through the degradation of textile pollutants, such as methylene blue (MB), rhodamine B (RhB), and eosin B (EB) [11]. Andoshe et al. have documented the synthesis of versatile TiO_2_ nanorods through a solution-based process. These nanorods were grown on a 4-inch p-silicon wafer, and they can be adjusted in terms of their heights and diameters. They have proven to be highly effective in splitting water when used as photocathodes [12]. Although there has been an enormous amount of research conducted on photocatalysts based on TiO_2_ nanorods, there is still a lack of understanding regarding the relationship between the morphology of TiO_2_ nanorods and their ability to catalyze reactions using light, especially when they are modified with Ag nanoparticles. Various morphological parameters, including the aspect ratio, surface area, and crystallographic orientation, can have a significant impact on photocatalytic activity. Hence, it is imperative to conduct a systematic investigation of the morphology-dependent photocatalytic activity of Ag-coated TiO_2_ nanorods in order to optimize their design for improved performance.

The aim of this study is to elucidate the influence of the morphology of Ag-coated TiO_2_ nanorods on photocatalytic activity. The novelty of this work is to study the impact of different morphological features on photocatalytic efficiency by synthesizing TiO_2_ nanorods, dense nanorods, needle-like nanorods, nanoballs, and nanoflowers and then decorating them with Ag nanoparticles. This research not only enhances our understanding of photocatalyst design but also facilitates the development of more efficient materials for harvesting solar energy and cleaning the environment.

## 2. Results

### 2.1. Titanium Dioxide Coating Characterization

**Figure 1:**
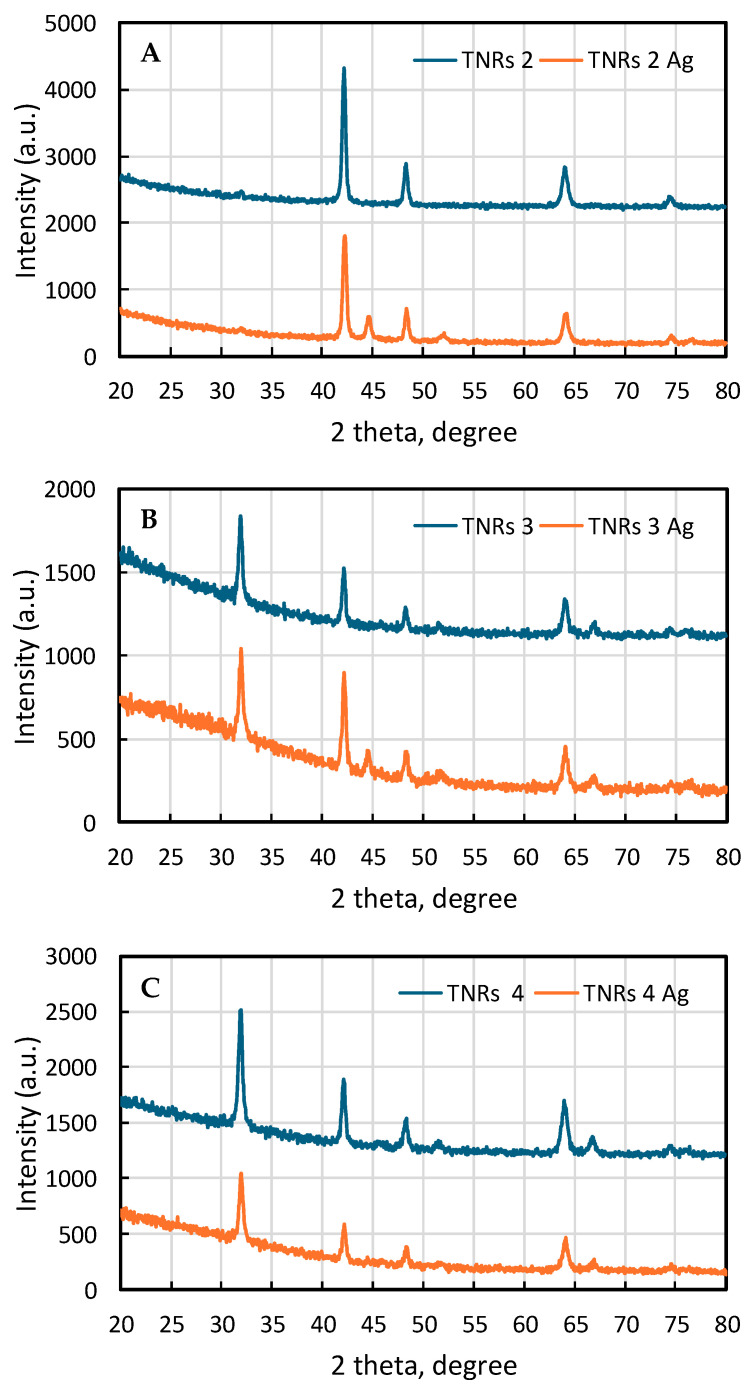
XRD patterns of (**A**) TNRs 2 and TNRs 2 Ag, (**B**) TNRs 3 and TNRs 3 Ag, and (**C**) TNR4 and TNRs 4 Ag nanostructures.

**Figure 2:**
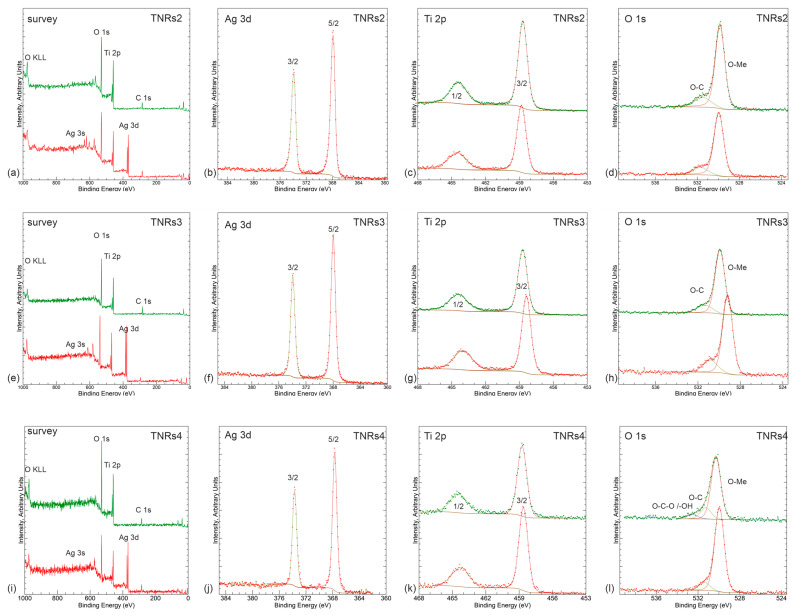
Results of XPS examination represented by wide survey scans and narrow high-resolution spectra for Ag 3d, Ti 2p, and O 1s regions obtained for TNRs 2 (panels (**a**–**d**)), TNRs 3 (panels (**e**–**h**)), and TNRs 4 (panels (**i**–**l**)) samples. For details, see the text. Green and red lines represent TNRs and TNRs modified with Ag, respectively.

**Figure 3:**
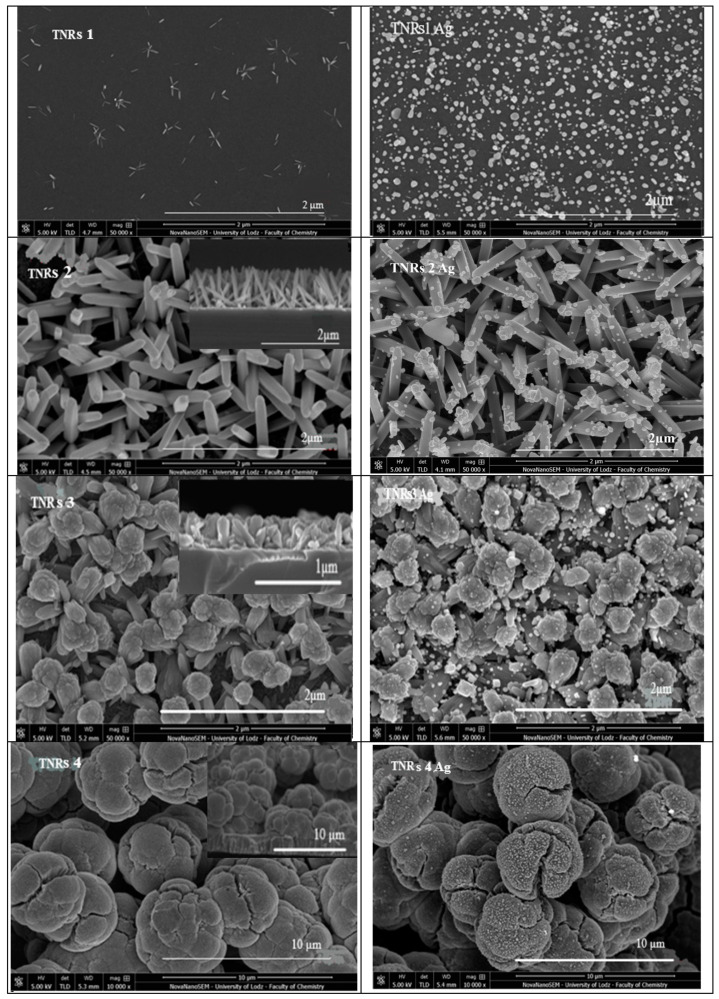

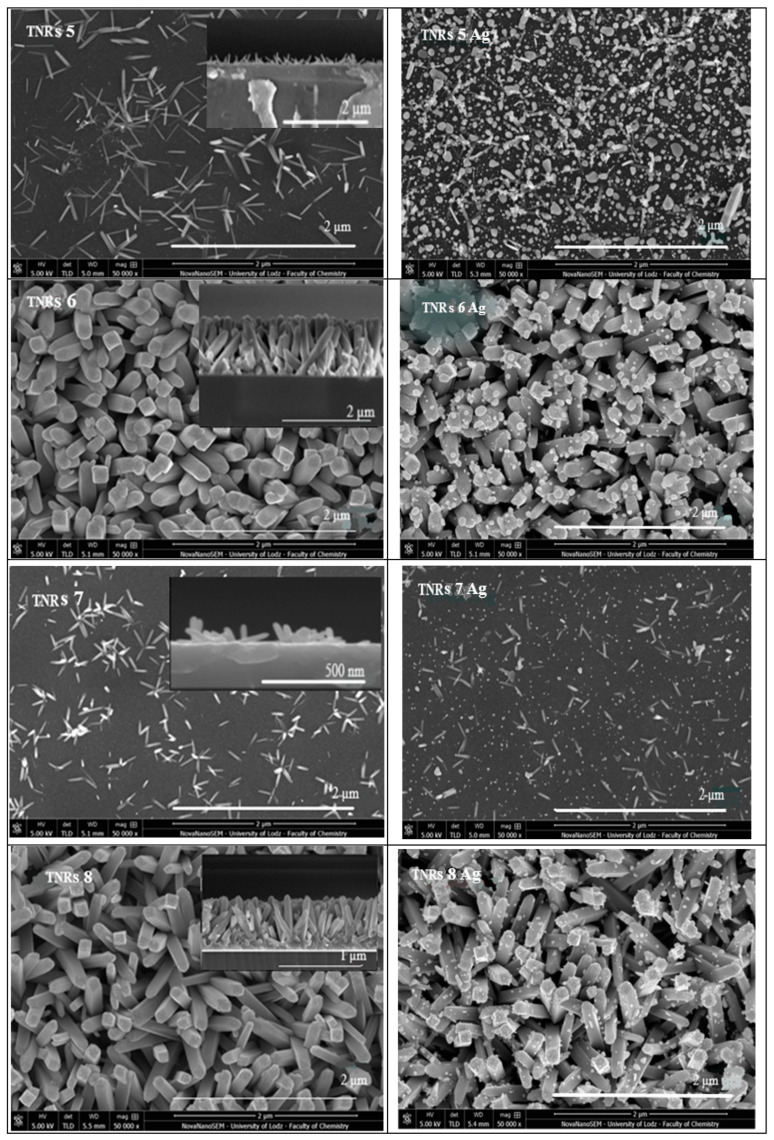
The SEM images of the TiO_2_ coating surface consisting of various nanostructures (**left**) and nanostructures modified with Ag (**right** column). The insets present a cross-sectional view of the TiO_2_ layer.

**Figure 4:**
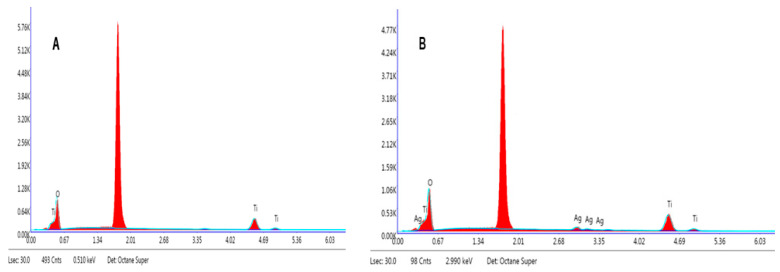
Chemical composition of the TNRs 2 (**A**) and TNRs 2 Ag (**B**) using energy-dispersive X-ray spectroscopy (EDX).

The main aim of the presented study was to show how the synthesis parameters affect the final morphology of the nanomaterials and, consequently, their photocatalytic properties. Upon changing such parameters, like the acid:water ratio, the amount of precursor, and the time of the reaction, the following morphologies were obtained and labeled: needle-like nanorods (TNRs 1, TNRs 5, and TNRs 7), nanorods (TNRs 2), nanoflowers (TNRs 3), nanoballs (TNRs 4), and dense nanorods (TNRs 6 and TNRs 8). The “TNRs” abbreviation refers to the whole family of materials with a basic morphology of titanium dioxide nanorods (TNRs). The main free morphologies, nanorods (TNRs 2), nanoflowers (TNRs 3), and nanoballs (TNRs 4), were thoroughly investigated in terms of the crystal structure (XRD) (Figure 1) and chemical states (XPS) (Figure 2). The SEM images of all obtained nanostructures along with the elemental analysis using EDS are presented in Figure 3 and Figure 4, respectively.

#### 2.1.1. X-ray Diffraction (XRD)

In Figure 1A–C, the XRD patterns of TNRs and Ag-modified TNR samples are presented. The XRD spectra of samples marked as TNRs 2, TNRs 3, and TNRs 4 show distinct peaks centered at 2theta around 31.98°, 42.16°, 48.26°, 51.63°, 64.02°, 66.85°, and 74.39°, which correspond to characteristic diffraction planes of rutile (Ref: 00-021-1276). Noteworthy is the fact that in the case of sample TNRs 2, there is a lack of peaks from the <110> and <210> crystalline planes (2theta 31.98° and 51.63°, respectively). This indicates the strong texture of the obtained TNRs with dominant <110> planes perpendicular to the normal to diffraction plane. Note that SEM pictures of those two TNRs may confirm this thesis. The diffraction pattern of sample TNRs 4 resembles a rather regular diffraction of the structure with a unidirectional orientation of coherent diffraction domains. Processes of subsequent surface modification of TNR samples resulted in the formation of crystalline silver nanoclusters. For all diffraction spectra of samples TNRs 2 Ag, TNRs 3 Ag, and TNRs 4 Ag, peaks coming from diffraction planes of silver may be visible: 2theta 44.57° and 51.9° (Ref: 00-004-0783). The intensity of the peaks decreases with increasing sample numbers, and for sample TNRs 4 Ag, they are barely noticeable, despite the fact that SEM EDX analysis confirmed the presence of silver in the chemical composition. Moreover, after the surface modification of all tested samples, the intensity of diffraction peaks of rutile noticeably decreased, indicating that the process of surface modification itself may affect the crystallinity of samples or may be the source of surface contamination by X-ray amorphous compounds.

#### 2.1.2. X-ray Photoelectron Spectroscopy (XPS)

XPS analysis was conducted to examine the surface chemical state of the Ti and O elements in TiO_2_. Figure 2a,e,i demonstrate the expected main core levels for TNRs 2, 3, and 4 samples, i.e., Ti 2p, O 1s, and, where applicable, Ag 3d. Slight carbonaceous residual contamination is also visible, as represented by C 1s. All panels show spectral lines for bare (upper lines) and Ag-modified samples (bottom lines). Further panels (with the same color code) present high-resolution spectra of Ag 3d, Ti 2p, and O 1s regions. Ag 3d regions present a near-metallic shape of 3d 3/2 and 5/2 components at the positions near the metallic state (i.e., 368.2 eV) [13]; however, they are slightly shifted towards an oxidized one by ~0.2 eV towards lower binding energies [14]. The narrow full width at half maximum (~0.6 eV) leaves no space for significant mixture of the phases here. The only divergence is in the case of the TNRs 4 sample, where the oxidation-related shift reaches 0.3 eV. For all samples, Ti 2p presents well-resolved peaks at ~458.8 eV and ~463.6 eV, which stand for Ti 2p_3/2_ and Ti 2p_1/2_, respectively. These peaks indicate the presence of Ti^4+^ in TiO_2_, as stated in reference [15]. The spin–orbit separation (~5.7 eV), high symmetry of components, and narrow FWHM (<0.9 eV for 2p_2/3_) indicate high stoichiometry of the oxide. According to a study conducted by Wang et al. [16], the binding energy of the Ti 2p core electron was found to be 457.1 eV for both the as-synthesized sample and after calcination at 450 °C. For our current sample, the binding energy of the Ti 2p electrons was measured to be 458.6 eV. This confirms the presence of Ti^4+^O_2_ as the primary titanium species, as supported by references [17,18,19].

In the case of TNRs 2, there is a shift of about 0.15 eV towards the lower binding energies for the layers with Ag, which might point to the partial existence of Ti^3+^’s contribution. For the TNRs 3, the shift almost reaches 0.4 eV, indicating more significant Ti^3+^ contribution, while for TNRs 4, the difference is negligible. The reduction of Ti^4+^ to Ti^3+^ is typically achieved through calcination [17]. The TiO_2_ nanoparticles were synthesized and then subjected to calcination at a temperature of 500 °C, resulting in the generation of Ti^3+^ ions. The panels (d), (h), and (l) in Figure 2 display the binding energy region of the O 1s in TiO_2_ nanostructures, which provides additional details regarding the elemental composition and oxidation states of the elements present in the Ag-TiO_2_ nanorods. The existence of one dominating lattice-related component is accompanied by a residual carbonaceous-related signal. No significant oxygen vacancy signal is detected, as indicated by a relatively small FWHM value once again (~1.0 eV). In the case of oxygen-related defects, the signal is overlapping with the carbonaceous component; however, having in mind carbon’s existence in the survey spectrum, there is a little space for vacancy contribution. In summarizing the XPS investigation, it is worth pointing out that the shifts of the Ag 3d and Ti 2p lines may not only be due to the contribution of different oxidation states but also due to interaction between TiO_2_ and Ag at the interface, as differentiated by various morphologies of the samples. This statement seems to be supported by topographical studies presented further. 

#### 2.1.3. Scanning Electron Microscope (SEM)

In order to further analyze the obtained coating, scanning electron microscopy (SEM) was performed. The SEM image in Figure 3 displays the coating surface composed of various nanostructures: needle-like nanorods (TNRs 1, TNRs 5, and TNRs 7), nanorods (TNRs 2), nanoflowers (TNRs 3), nanoballs (TNRs 4), and dense nanorods (TNRs 6 and TNRs 8). The left column presents unmodified materials, while the same materials modified with Ag are in the right column. These morphologies are the result of conducting the synthesis at various preparation parameters. Figure 3 also depicts a cross-sectional view of the TiO_2_ layer that has been grown on sol–gel TiO_2_ coating deposited onto the silicon wafer. The layer has a consistent thickness throughout its entire length. The elemental composition of the Ti, O, and Ag nanoparticles was determined using energy-dispersive X-ray (EDX) analysis. The EDX spectra of TNRs along with TNRs Ag, as illustrated in Figure 4, exhibit distinct peaks corresponding to the Ag, Ti, and O constituents, indicating the successful coating of Ag nanoparticles with a TiO_2_ layer. Other EDX spectra for the investigated nanostructures exhibit similar signals. The detailed EDX analyses of all of the samples are shown in Table 1.

### 2.2. Synthesis of TiO_2_ Nanorods on Si/TiO_2_ Substrate

#### 2.2.1. Effect of the Acid:Water Ratio

It was reported that for a volume ratio of HCl:H_2_O of 45:45 mL, mostly nanorods were observed. Meanwhile, a nanoball-like structure was formed for a volume ratio of HCl:H_2_O ranging from 50:40 to 20:70. Hence, in order to optimize the HCl:H_2_O volume ratio, the nanomaterials labeled TNRs 1, TNRs 2, TNRs 3, and TNRs 4 (corresponding to HCl:H_2_O ratios of 50:40, 45:45, 25:65, and 20:70 mL, respectively) were compared and modified with Ag. SEM images in Figure 3 clearly show that nanoflowers and nanoballs composed of tiny nanorods were formed for both the TNRs 3 and TNRs 4 samples. For the TNRs 1 and TNRs 1 Ag, a lower density of nanorod-like structures was observed. The average length of the nanorods of TNRs 1 was 83 ± 20 nm, and the average diameter was 13 ± 5 nm. The average diameter of the Ag particles of TNRs 1 Ag was 48 ± 23 nm, as shown in Table 2. For the sample TNRs 2, the length of the nanorods and the diameter were 863 ± 50 and 97 ± 22 nm, respectively. Also, upon comparing the diameters of the nanoflowers on TNRs 3 and the Ag on TNRs 3 Ag, the diameters were in the range of 285 ± 44 and 35 ± 20 nm, respectively, and the length was 634 ± 57 nm, whereas for samples TNRs 4 and TNRs 4 Ag, the diameter of the nanoball was in the range of 4279 ± 416, and the size of the Ag was 50 ± 36 nm, while the length was 2064 ± 142 nm. According to Prathan et al., the mechanism of formation of TiO_2_ nanorods is based on the four main steps [20]. The first part is hydrolysis, where butyl groups (-OBu) are replaced by hydroxyl groups (-OH). The second part involves protonation of Ti-OH groups by HCl and becoming Ti-OH_2_^+^ groups that combine with the -OH groups of other TiO_6_. In a third step called olation, the process of dehydration or condensation occurs, which combines intermediate compounds to form metal oxides and release water molecules. Olation occurs in highly acidic mediums producing long chains of highly protonated Ti complexes. Finally, in the oxolation step, the formation of larger NRs occurs and contributes to the development of a lateral arrangement in less-protonated solutions. In our reactions, the high concentration of HCl causes high protonation, which in consequence leads to fast olation and results in long chains of TiO_6_ octrahedra. For low or insufficient HCl concentrations, nanorods are not created, and other types of nanostructures, like nanoflowers (branched nanorods) and spherical nanostructures (nanoballs), are formed.

#### 2.2.2. Effect of the Precursor Amount

The titanium (IV) butoxide (TTBO) precursor acts as a source of crystal growth after nucleation. Liu et al. [6] reported that the presence of TTBO in the mother solution affects the size and diameter of nanostructures. Thus, in order to reduce the rate of deposition and to slow the reaction kinetics, a synthesis of samples TNRs 5 (TTBO = 1 mL) and TNRs 6 (TTBO = 2 mL) was executed, and the samples were analyzed sequentially. The SEM of sample TNRs 5 (Figure 3) and modified with silver TNRs 5 Ag for morphological examination showed a needle-like structure. These nanorods had an average diameter in the range of 23 ± 9 nm, with a length of 236 ± 33 nm of TNRs 5, and the diameter of the Ag of TNRs 5 Ag was 26 ± 17 nm. However, the SEM of samples TNRs 6 and TNRs 6 Ag showed the formation of dense nanorods on the TiO_2_ substrate. The nanorods had an average diameter in the range of 130 ± 18 nm, with a length of 929 ± 74 nm, and the diameter of the Ag of TNRs 6 Ag was 33 ± 20 nm. Thus, by reducing the volume of the precursor TTBO, the amount of titanium dioxide precursor available for hydrolysis and condensation reactions was reduced. This, in turn, reduced the rate of aggregation of nanorods to yield a needle-like structure. Hence, the parameters controlling the reaction kinetics of hydrolysis and growth can be altered by the amount of the precursor TTBO.

#### 2.2.3. Effect of the Reaction Time

To understand the impact of parameters that regulate the reaction kinetics, we synthesized nanomaterials labeled TNRs 2, TNRs 7, and TNRs 8, which were modified subsequently with Ag and analyzed. Nayak et al. [21] found that the time parameters involved in the reaction have a crucial role in determining the kinetics of the reaction, which affects the size of the nanostructures and the rate at which they are deposited. Below a reaction time of 4 h, growth was exceptionally slow. An elongation of the nanostructure was observed after exceeding a duration of 4 h. Furthermore, three distinct samples, namely TNRs 2, TNRs 7, and TNRs 8, were produced through synthesis at a temperature of 150 °C for durations of 4, 2, and 6 h, respectively, and modified with Ag. The SEM for samples TNRs 7, TNRs 8, TNRs 7 Ag, and TNRs 8 Ag, as depicted in Figure 3, demonstrates the impact of time on the formation of nanostructures at a morphological level. The SEM analysis revealed that TNRs 7 (short time of 2 h) had a lower density of nanorod-like structures, which were observed to be less densely packed, and it had angular rods that were growing freely. The diameter of sample TNRs 7 was 20 ± 6 nm, while the length of the nanorods was 117 ± 30 nm. The diameter of the Ag on the sample TNRs 7 Ag was 20 ± 6 nm. Therefore, by decreasing the time it takes for a reaction to occur, it is possible to regulate the speed at which nanorods come together and form clusters. Nevertheless, the scanning electron microscopy (SEM) analysis of sample TNRs 8 (long time of 6 h), as depicted in Figure 3, revealed the presence of tightly packed and elongated nanorods on the TiO_2_ substrate. The nanorods exhibited an average diameter of 104 ± 21 nm, with a length of 1133 ± 124 nm. Therefore, based on the aforementioned observations, it can be inferred that the arrangement of nanorods in space becomes more loosely packed as time decreases, which is the effect of a decrease in the rate at which TiO_2_ nanostructures are deposited. The average dimensions of the whole family of TNR materials are presented in Table 2.

#### 2.2.4. Effect of the Morphology on Ag NPs’ Size

Regarding the size of AgNPs grown on the TNRs, it can be noticed that their size is changing in the range of 16–50 nm and depends on the morphology of prepared materials. The smallest average size of Ag NPs (d = 16 nm) exhibits a TiO_2_ surface with a small amount of short TiO_2_ nanorods (TNRs 7). In the case of a group of photocatalysts with similar morphologies, i.e., TNRs 8, 5, 6, 2, and 3, the AgNPs’ size is comparable and equals 24, 26, 33, 34, and 35 nm, respectively. One of the largest sizes of AgNPs (d = 48 and 50 nm) was recorded for the coatings with the smallest, rarely distributed nanorods (TNRs 1) and for round-shaped, cauliflower-like materials (TNRs 4). Although the exact mechanism explaining the differences in growth and subsequent size of AgNPs on individual types of coatings (TiO_2_ crystals) requires further study, it is clear that one of the key parameters affecting the AgNPs’ size is the morphology of prepared coatings (Table 2).

### 2.3. Determination of the Band Gap Eg

The band gaps of prepared materials were determined from optical measurements and calculated according to the Tauc method (details are in the experimental section). It was found that for materials for which the effect of the acid:water ratio (TNRs 1, TNRs 2, TNRs 3, and TNRs 4, corresponding to HCl:H_2_O ratios of 50:40, 45:45, 25:65, and 20:70 mL, respectively) was investigated, the value of the Eg energy level was in the range of 3.34 to 2.96 eV. These values were lowered after modification with AgNPs.

**Figure 5:**
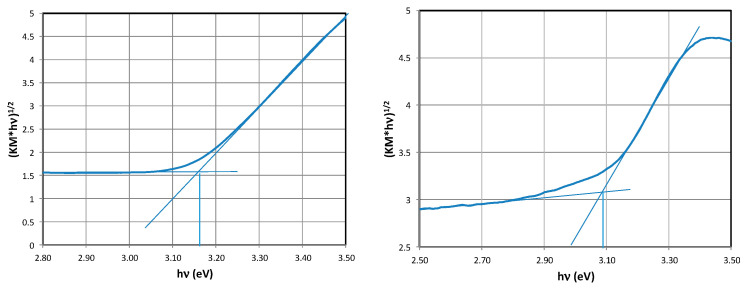
Tauc plots of the TNRs 2 and TNRs 2 Ag materials.

Increasing the amount of the TTBO precursor (1.0 mL, 1.5 mL, and 2 mL, corresponding to TNRs 5, TNRs 2, and TNRs 6) results in a decrease in the band gap, which is additionally amplified by the presence of silver. A similar effect of lowering the Eg energy was obtained for materials prepared under increasing hydrothermal synthesis time (2 h, 4 h, and 6 h, corresponding to TNRs 7, TNRs 2, and TRNs 8), which was also additionally enhanced by the presence of AgNPs. Details concerning the Eg measurements are presented in Table 3. Representative results showing the determination of Eg values with the use of a Tauc plot are shown in Figure 5. For all other materials, the Eg values were determined using the same method.

### 2.4. Photocatalytic Decomposition of Rhodamine B (RhB)

The photocatalytic properties of the obtained photocatalysts were evaluated by observing changes in the RhB solution spectrum during irradiation. Experiments were conducted using an aqueous solution of RhB in two different ranges of light: (i) ultraviolet (UV) and (ii) visible (Vis). This study focused on two distinct phenomena: the direct activation of a photocatalyst and sensitization. The UV light triggers the activation of TiO_2_, resulting in the production of hydroxyl radicals (^•^OH) and other forms of reactive oxygen. These species undergo a reaction with the RhB molecules, resulting in their decomposition [22,23]. However, visible light does not stimulate the TiO_2_, but it can impact the RhB molecules, causing them to become excited. Subsequently, oxygen radicals are formed on the surface of TiO_2_ through an indirect sensitization process. This occurs when electrons are transferred from the lowest unoccupied molecular orbital (LUMO) of RhB molecules in an excited state to the conduction band of TiO_2_ [24]. This is extensively utilized in the domains of dye-sensitized solar cells (DSSCs) and photocatalysis [25,26]. These two modes of stimulation lead to distinct routes of RhB decomposition.

**Figure 6:**
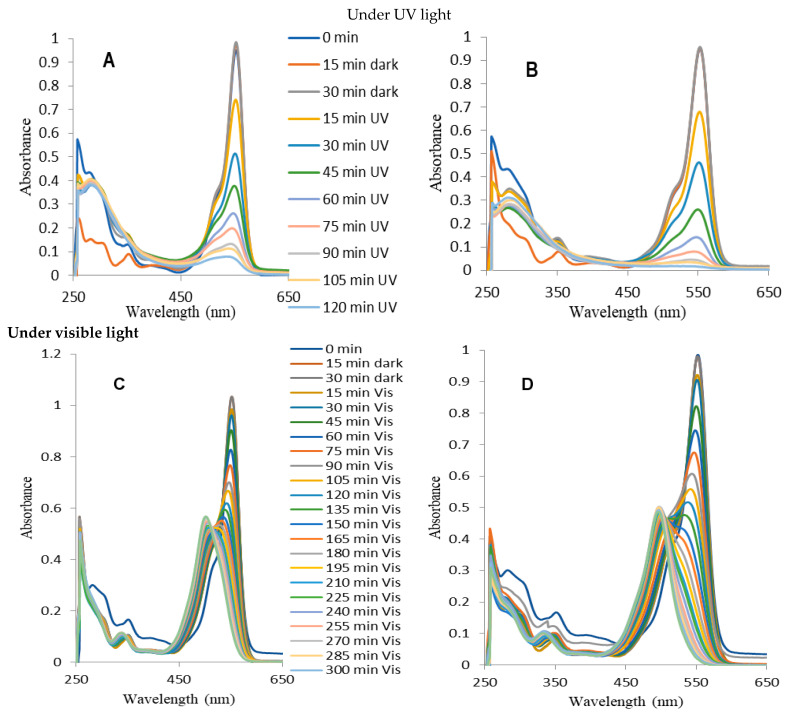
Graphs illustrating the alterations in the absorption spectrum of RhB when exposed to UV ((**A**) TNRs 2 and (**B**) TNRs 2 Ag) and visible light ((**C**) TNRs 1 and (**D**) TNRs 1 Ag).

Figure 6 shows compiled graphs illustrating the alterations in the absorption spectrum of RhB when exposed to UV and visible light in the presence of TNRs and TNRs modified with Ag. When exposed to UV irradiation, there is a noticeable decrease in the peak height at around 554 nm, while the positions of the peaks remain relatively unchanged. This is a common occurrence when the chromophore undergoes destruction during the process of RhB photodegradation. The determination of the reaction rate constant (k) based on Langmuir–Hinshelwood’s model (Equation (1)), following the pseudo-first-order kinetics expressed by [27], involved measuring the absorbance value at a wavelength of 554 nm.

ln(C/C_0_) = −kt (1)

**Figure 7:**
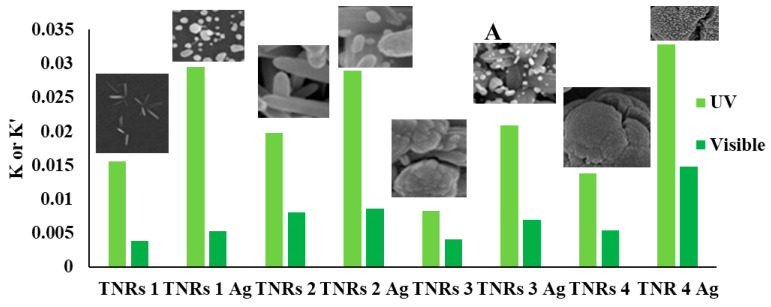

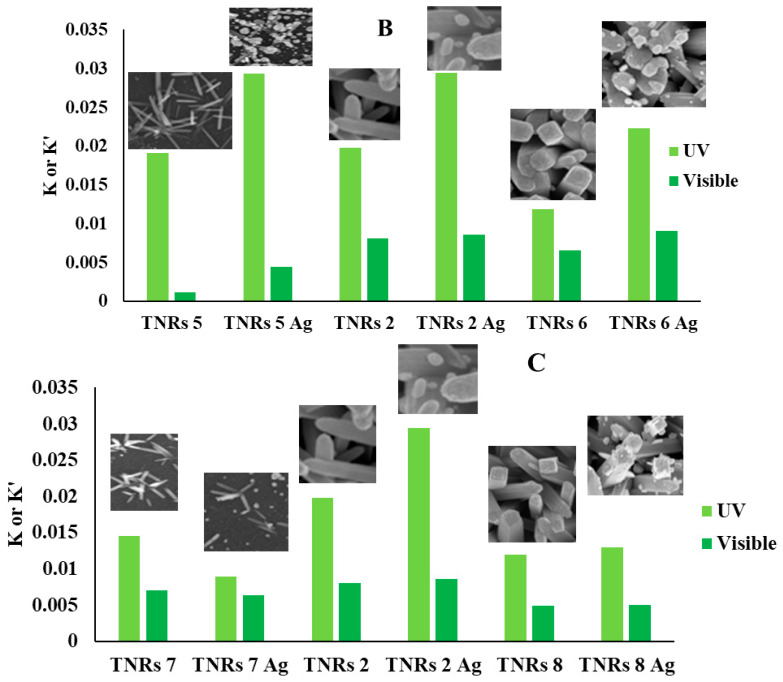
Graphical representation of decomposition rate constants k and k′ for different parameters of nanomaterials preparation. (**A**) Effect of chemical composition; (**B**) effect of precursor amount; (**C**) effect of reaction time.

The equation represents the relationship between the pseudo-first-order rate constant (k (min^−1^)), the initial concentration of RhB (C_0_), and the concentration of RhB at a given time (C). The graphical representations of k (for UV) and k′ (for visible) are shown in Figure 7. This methodology is frequently employed when estimating the activity of photocatalysts in dye photodecomposition [28,29,30,31,32,33]. However, when evaluating the activity of a photocatalyst under visible light irradiation, the reaction rate constant parameter alone is not enough. During prolonged exposure to visible light, the initial absorbance value at the peak wavelength of 554 nm decreases. Simultaneously, the hypochromic shift becomes apparent, and after 285 min of the photocatalytic process, the peak centered at 498 nm is isolated. After a duration of 285 min, the spectrum remains unchanged. The highest point at 498 nm was determined to be a signal originating from rhodamine 110 (Rh-110), which aligns with findings from previous research on the photocatalytic conversion of rhodamine B in the presence of various substances, such as TiO_2_ [34,35,36], TiO_2_-Ag [34], TiO_2_-Si [35], and TiO_2_-GO-Ag [37], under visible light irradiation. Identical outcomes were noted during photocatalytic measurements conducted under visible light irradiation for each type of coating examined in this study.

Spilarewicz-Stanek et al. [37] described the photocatalytic transformation of RhB into Rh-110 using the N-deethylation mechanism. During this procedure, the amino diethyl groups of RhB undergo elimination of ethyl groups, leading to the sequential formation of N,N,N′-triethyl-Rh-110, N,N′-diethyl-Rh-110, N-ethyl-Rh-110, and Rh-110. These compounds exhibit maximum absorption at wavelengths of 539 nm, 522 nm, 510 nm, and 498 nm, respectively. The presence of ethyl groups in the structure of RhB molecules serves as auxochromes, which determines the precise location of the absorption maximum. Their removal results in the hypochromic shift observed in the spectrum. It was discovered that when ethyl groups are removed from the dye that is adsorbed on the surface of the photocatalyst, the extra negative charge that builds up on the TiO_2_ surface is eliminated by the oxygen molecule that is adsorbed, resulting in the formation of the superoxide radical [38]. In this scenario, indirect sensitization alters the reaction pathway of reactive oxygen species (ROS) generation, leading to an increase in the production of superoxide radicals O_2_^•−^ and a decrease in the likelihood of ^•^OH formation. To mathematically estimate this phenomenon, our study proposes the determination of the conversion efficiency factor (Wc) (Equation (2)), shown in Table 4. This parameter is determined by the ratio of the actual number of moles (n_a_) of the substance produced through a chemical reaction to the theoretical number of moles (n_t_) of the same substance that would be obtained if all of the starting molecules were converted into product. The formula describes the conversion efficiency.

W_c_ = n_a_/n_t_ × 100% (2)

Because the chemical conversion of RhB to Rh-110 occurs within the same volume, it is possible to convert the number of moles into a molar concentration. The formula is transformed into the following expression after applying the Bouguer–Lambert–Beer law (Equation (3)):

W_c_ = [(A_Rh-110_ × ε_RhB_)/(A_RhB_ × ε_Rh-110_)] × 100% (3)

To perform the necessary calculations, the molar extinction coefficient (ε) for RhB and Rh-110 was determined through experimental estimation. The value of RhB was determined to be 89,590 cm^−1^ M^−1^, while the value of Rh-110 was found to be 70,605 cm^−1^ M^−1^. The current study involved the calculation of the conversion coefficient by utilizing the A_RhB_ value at the maximum peak wavelength of 554 nm from the initial RhB solution (with a concentration of 1 × 10^−5^ M). Additionally, the A_Rh-110_ value at the maximum peak wavelength of 498 nm was measured after 285 min of the photocatalytic process. The determination of conversion efficiency enables the calculation of removal efficiency (W_r_) (Equation (4)), as well.

W_r_ = 100% − W_c_ (4)

Removal efficiency refers to the extent to which RhB molecules are degraded through photocatalytic processes, leading to the destruction of chromophores. When studying photocatalytic performance, it is important to analyze the conversion efficiency and the removal efficiency together with the reaction rate constant, rather than considering them separately. Figure 7 demonstrates that all of the nanostructures that have been coated with silver, with the exception of TNRs 7 Ag, are more effective than the samples that do not have any coating. The modification of nanoball-like structures with silver (TNRs 4 Ag) demonstrated high photocatalytic efficiency under both ultraviolet (UV) and visible light for different chemical composition parameters. However, for the precursor amount parameter, the TNRs 2 Ag sample (nanorod structure) exhibits higher efficiency under UV, while the TNRs 6 Ag sample is more effective under visible light for the photocatalyst activity. In terms of the time parameter, the TNRs 2 Ag sample exhibits a higher level of activity for both ultraviolet (UV) and visible light. Table 4 presents the reaction rate constants of TNRs and TNRs Ag for UV and Vis light, including the calculation of conversion efficiency for RhB transformation under visible light.

The graphs presenting the effects of individual nanostructures affecting the photocatalytic properties are presented in Figure 7.

## 3. Discussion

### 3.1. Transformations of RhB under Visible Light

The production of Rh-110 through visible light irradiation of TNRs and TNRs Ag is a unique phenomenon. The photocatalytic transformation of RhB to Rh-110 in the presence of TNRs can be readily elucidated by the chemisorption mechanism when exposed to visible light. This mechanism is based on the following chemical reactions, which comprise the excitation of RhB, the transfer of electrons from RhB to TiO_2_, the formation of ROS, and, finally, the transformation of RhB into decomposition products [39,40].

RhB + hv → RhB*

RhB* + TiO_2_ → ^•^RhB^+^ + TiO_2_ (e^−^)

TiO_2_ (e^−^) + O_2_ → ^•^O_2_^−^

^•^RhB^+^ + ROS (^•^O_2_^−^) →→ RhB110

^•^O_2_^−^ + H^+^ → OOH*

^•^OOH + ^•^O_2_^−^ + H^+^ → O_2_ + H_2_O_2_

H_2_O_2_ + ^•^O_2_^−^ → ^•^OH + OH^−^ + O_2_

^•^RhB^+^ + ROS (^•^OH) → Degraded products

**Figure 8:**
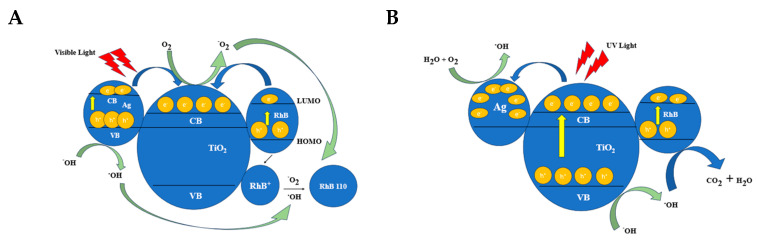
Proposed mechanism for the photocatalysis of RhB in the presence of TNRs modified with Ag under (**A**) visible and (**B**) UV light.

The chemisorption mechanism of RhB with various functional groups explains the photocatalytic oxidation results for TNRs 1 to TNRs 8 when exposed to visible and UV light (Figure 6) [35]. Due to variations in the surface morphology and crystallinity of TNRs 1 to TNRs 8, the carboxyl (-COOH) or diethylamino (-N(C_2_H_5_)_2_) groups of RhB can selectively adhere to the surface of TiO_2_. During light irradiation, the carboxyl (-COOH) group of RhB chemisorbs onto the photocatalyst’s surface, leading to the destruction of the conjugated xanthene structure. In contrast, when RhB chemisorbs with -N(C_2_H_5_)_2_, Rh-110 is formed [41]. The AgNPs facilitate the conversion of RhB to Rh-110 by capturing electrons. The electrons confined within metallic centers (AgNPs) undergo a reaction with adsorbed oxygen, resulting in the generation of superoxide radical ^•^O_2_^−^. This radical facilitates the conversion of RhB into Rh-110. Furthermore, previous studies have suggested that the N-deethylation of RhB primarily takes place on the surface through the action of ^•^OH radicals [42]. However, it is worth noting that a typical TiO_2_ surface, despite having a short lifespan of ^•^OH radicals, can also generate Rh-110 when exposed to UV radiation [41]. This implies that the presence of other radicals, in addition to ^•^OH radicals, may also play a significant role in the formation of Rh-110. Based on the mechanism of photosensitized degradation and the chemisorption of RhB, we hypothesize that the formation of Rh-110 may be influenced by the quantity and type of radical species. A potential mechanism for the selective oxidation of TNRs Ag is depicted in Figure 8. As depicted in Figure 8, the wide band gap of TiO_2_ prevents the formation of photoinduced electron–hole pairs in TiO_2_ when exposed to visible light. Nevertheless, the localized surface plasmon resonance (LSPR) of silver nanoparticles (Ag NPs) can generate photoinduced electron–hole pairs on the Ag NPs’ surface. Simultaneously, RhB can also generate photoinduced electron–hole pairs on its lowest unoccupied molecular orbital (LUMO) and highest occupied molecular orbital (HOMO) [43] due to photosensitization. The energetic electrons can be transferred to the conduction band (CB) of TiO_2_ as a result of the strong collective oscillation of electrons upon LSPR excitation [44,45]. The outcome is that the surface of TiO_2_ exhibits a diverse range of electronic states. This is due to the presence of ^•^O_2_^−^ on the surface of TiO_2_, which is formed as a result of the dissolved oxygen in water. Additionally, ^•^OH can be acquired by means of ^•^O_2_^−^ [40]. However, due to the enhanced electron supply from the photosensitized RhB, the impact of ^•^O_2_^−^ is greater than that of ^•^OH in this particular scenario, resulting in the production of Rh-110 under visible irradiation. The TNRs 4 Ag, TNRs 6 Ag, and TNRs 2 Ag exhibited excellent photocatalytic efficiency under visible light due to their relatively large surface area compared to other TNR samples. Additionally, the presence of AgNPs facilitated the conversion of RhB to Rh-110 through electron trapping.

### 3.2. Decompositions of RhB under UV Irradiation

The silver modification of nanoball-like structures (TNRs 4 Ag) exhibited a significant increase in photocatalytic efficiency under ultraviolet light for different chemical composition parameters. However, when considering the precursor amount parameter, the TNRs 2 Ag sample (nanorod structure) demonstrates superior efficiency in the ultraviolet range for photocatalyst activity. The TNRs 2 Ag sample demonstrates a greater level of activity under ultraviolet in terms of the time parameter. When exposed to UV radiation (as shown in Figure 6), pure TiO_2_ can produce electron–hole pairs through a photoinduced process. The conduction band (CB) energy level of TiO_2_ is greater than the Fermi energy level of Ag. This allows photo-excited electrons to transfer from TiO_2_ to Ag NPs due to the potential energy difference. Ag NPs serve as an electron sink [44]. In other words, the recombination of photo-induced electrons and holes is decreased, resulting in an extended lifespan. In this scenario, electrons that have been excited by light can be readily captured by dissolved O_2_ and H_2_O, resulting in the formation of ^•^OH on the surface of Ag NPs [46]. The h^+^ of TiO_2_ can also be captured by -OH, which primarily originates from water molecules adsorbed on the surface of TiO_2_, resulting in the formation of ^•^OH. Under UV irradiation, the impact of ^•^OH is more significant than that of ^•^O_2_^−^. Due to the greater impact of ^•^OH compared to ^•^O_2_^−^ when exposed to UV radiation, the conjugated xanthene structure of RhB is disrupted, preventing the formation of Rh-110 under UV light. This conjecture can be further elucidated by considering the oxidation potential of radicals. The oxidation potential of ^•^OH is commonly acknowledged to be 2.80 V [39] (or 2.7 V [47]), while molecular oxygen (or H_2_O_2_) has an oxidation potential of 1.76 V [48]. The reduction potential of O_2_ (O_2_/^•^O_2_^−^) is −0.33 V [47], and the oxidation potential of the photo-excited RhB (RhB*) is −1.40 V [47] (or −1.09 V [49]). This indicates that reactive oxygen species (ROS) exhibit a higher level of positivity compared to the excited state of Rhodamine B (RhB). As a result, the process of photocatalytic oxidative degradation of RhB can occur more readily through the action of ROS. However, this also indicates that the conjugated xanthene structure of RhB could not be disrupted by ^•^O_2_^−^, as the potential of ^•^O_2_^−^ is lower than that of ^•^OH. During the photocatalytic oxidation process, the RhB molecule’s conjugated xanthene structure can undergo a reaction with ^•^O_2_^−^ to initially produce a temporary state when exposed to visible light. The transient state serves to protect the conjugated xanthene structure of RhB, allowing only the N-deethylation reaction of RhB to occur through the action of ^•^OH, resulting in the formation of Rh-110 [34]. Contrarily, the impact of ^•^OH is more significant than ^•^O_2_^−^ when exposed to UV radiation, resulting in a shorter duration of the transient state. Consequently, Rh-110 cannot be achieved. The unusual deviation in the behavior of TNRs 7 Ag can be attributed to the substantial accumulation of Ag on the surface of the TiO_2_ nanorods, which obstructs the active sites that are crucial for photocatalytic reactions (as demonstrated in Table 1). Excessive coverage of the TiO_2_ surface by Ag leads to a decrease in the total surface area available for catalytic reactions. The mechanism of RhB decomposition under UV illumination can be described by the following chemical reactions: excitation of TiO_2_, electron trapping by Ag or/and O_2_, and degradation of RhB with ^•^OH radicals.

TiO_2_ + *hv* → TiO_2_ (e^−^ + h^+^)

TiO_2_ (e^−^)+ AgNPs → TiO_2_ + AgNPs (e^−^)

AgNPs (e^−^) + O_2_ → ^•^O_2_^−^ → ^•^OH

TiO_2_ (e^−^) + O_2_ → ^•^O_2_^−^ → ^•^OH

TiO_2_ (h^+^) + H_2_O → TiO_2_ + ^•^OH + H^+^

RhB^+^ + ROS (^•^OH) → Degraded products

The potential mechanisms of TNRs’ excitation and the mechanisms of RhB decomposition/transformation under visible and UV light are presented in Figure 8.

## 4. Materials and Methods

### 4.1. Materials

Silicon wafers exposing the (100) surface (ITME—Institute of Electronic Materials Technology, Warsaw, Poland) were selected as a substrate for TiO_2_ thin coating preparation. Reagents for sol preparation were titanium tetraisopropoxide (Sigma Aldrich, currently Merck/MilliporeSigma, Burlington, MA, USA 99.7%), isopropanol (Avantor Performance Materials, Gliwice, Poland S.A., pure, min. 99.7%), and hydrochloric acid (37%, Merck/MilliporeSigma, Burlington, MA, USA). The titanium (IV) butoxide (TTBO, 98%, Merk) was used for the synthesis of TiO_2_ nanorods. Silver nitrate (Avantor Performance Materials Poland S.A., 99.85%) solution at a concentration of 0.1 mM was prepared in ethanol (Avantor Performance Materials, Gliwice, Poland S.A., pure, min. 99.7%). Rhodamine B (Sigma Aldrich, currently Merck/MilliporeSigma, Burlington, MA, USA, pure ~95%) solution at a concentration of 1 × 10^−5^ M was prepared in deionized water. Deionized water was obtained through purification using the Millipore Simplicity UV system (18.2 MW cm at 25 °C, New York, NY, USA). Photocatalytic experiments were carried out in PMMA disposable cuvettes (ROTH, Rotilabo^®^, Disposable Cells No. A-208, 4.5 mL, 10 mm × 10 mm Light Path, four optically clear sides).

### 4.2. Preparation of Titanium Dioxide Coatings

Firstly, the silicon substrates were cleaned in a mixture of ethanol and isopropanol in an ultrasonic bath, then with a dust-free cloth moistened with ethanol, and, lastly, with a stream of compressed air. Titanium dioxide coatings were obtained using the sol–gel method reported in [34] and in our previous studies [50,51]. Briefly, in the typical procedure of the sol–gel method, 1.246 mL (0.00422 mol) of titanium tetraisopropoxide and 0.0399 mL of 2 M (0.0011 mol) hydrochloric acid were added dropwise to 16.92 mL of isopropanol (0.220 mol), and the mixture was stirred for 30 min at room temperature. Furthermore, prepared sol was applied on silicon wafer substrates using the dip-coating method by using a computer-controlled dip-coater (NIMA Technology LB, Coventry, UK). Dipping at an immersion–withdrawal velocity of 25 mm/min was repeated two times while preserving a drying time of 15 min between repetitions. Obtained coatings were annealed at 100 °C and calcined at 500 °C (both for 2 h), leading to obtaining an anatase phase.

### 4.3. Hydrothermal Growth of TiO_2_ Nanorods

#### 4.3.1. Pre-Treatment of the Substrate

The silicon wafer Si (3 × 0.9 cm) with a TiO_2_ double layer was cleaned with detergent, deionized water, acetone, and ethanol (1:1:1) in sequence and sonicated for 30 min at room temperature. After, it was washed with deionized water 3 or 4 times.

#### 4.3.2. Synthesis of TiO_2_ Nanorod

The deionized water, DI water, and HCl (37%, Merck) were mixed (45 mL:45 mL) during 10 min of magnetic stirring accompanied by the addition of 1.5 mL of titanium(IV) butoxide (TTBO, 98%, Merk) until a clear solution was obtained. Afterwards, the mixture was transferred to a 100 mL Teflon-lined stainless-steel autoclave. The silicon wafers were placed vertically at an angle of 45–60° and heated at 150 °C for 4 h to achieve the TNRs on the cleaned TiO_2_ double-layer silicon wafers. Eventually, the autoclave was cooled to room temperature. The synthesized photocatalysts were collected and washed three times with distilled water and then dried at 100 °C for 1 h. Finally, the substrates were calcined at 500 °C for 1 h to obtain TNRs arrays. The different combination schemes for synthesis are shown in Table 5.

### 4.4. Surface Modification of TiO_2_ Nanorods with Silver Nanoparticles (AgNPs)

**Figure 9:**
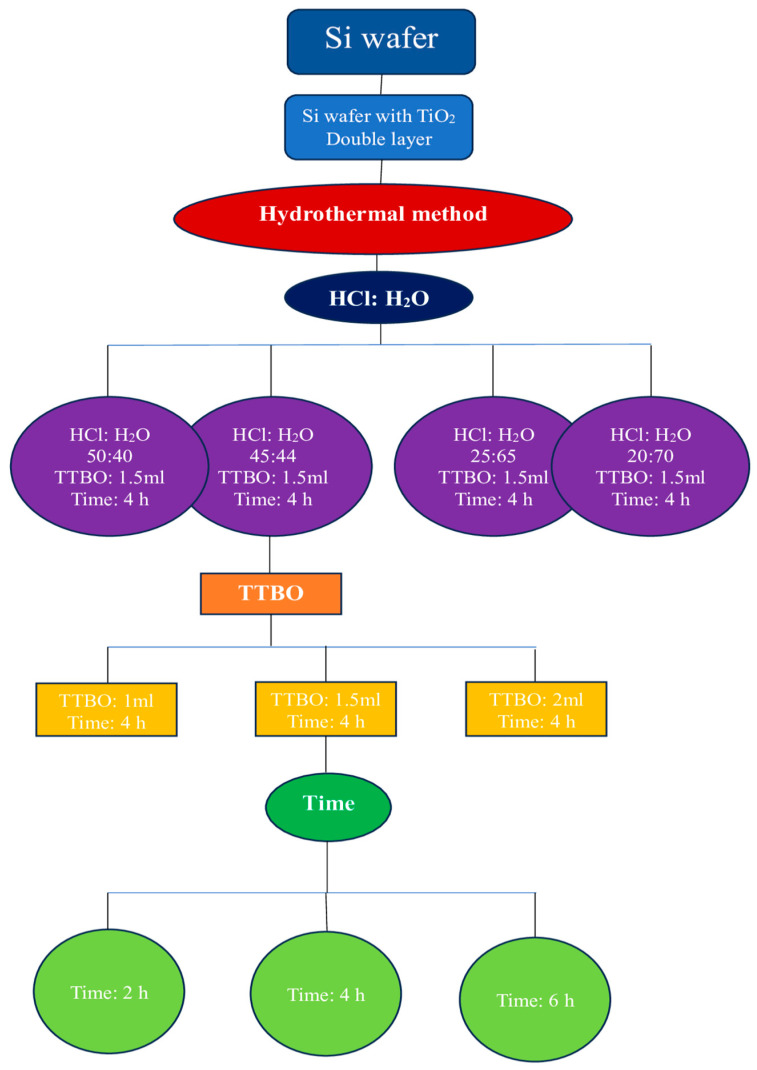
Flowsheet diagram of TNRs scheme preparation. Obtained materials were subsequently modified with AgNPs deposited in photoreduction of Ag^+^ ions with the use of UV light. (UV-Consulting Peschl, wavelength λ = 365 nm, t = 5 min.).

Silver nanoparticles (AgNPs) on the photocatalysts’ surfaces were obtained through photoreduction of Ag^+^ ions. Therefore, AgNO_3_ in ethanol and deionized water (1:1) solution at a concentration of 1 mM was used as a precursor. Previously prepared coatings (TiO_2_ nanorods) were immersed in polymethacrylate (PMMA) cuvettes containing 3.5 mL of AgNO_3_ ethanol and deionized water (1:1) solution in the dark for 30 min. Then, for 5 min, the cuvettes were irradiated with a UV lamp (UV-Consulting Peschl, wavelength λ = 365 nm). The power density measured on the sample level was 5 mW cm^−2^. The angle between the surface of the coatings and the UV rays was set at 90°. The distance between the samples and the light source was fixed at 20 cm. Subsequently, the coatings were rinsed with deionized water to remove residual ions/solution. The flowsheet diagram of TiO_2_ preparation is presented in Figure 9.

## 5. Characterization

The X-ray diffraction studies were conducted on an Empyrean diffractometer (Panalytical) working with Co Kα radiation (λ = 0.1780 nm) and GIXRD mode with an incident beam angle of 1°. The obtained data were processed using HighScore Plus with the ICDD PDF 5 + Database.

### 5.1. XPS Measurements

The XPS experiment was conducted in an ultra-high-vacuum experimental setup (base pressure 8.5 × 10^−11^ mbar) with the use of a PREVAC EA15 hemispherical electron energy analyzer (fitted with 2D-MCP detector) and a monochromated X-ray source (PREVAC dual-anode XR-40B source, RMC50 monochromator). A Al-Kα excitation line (energy 1486.60 eV) was used for sample irradiation. Pass energy (PE) of 200 eV (scanning step of 0.9 eV) was used for survey spectra collection, while 100 eV was used for high-resolution energy regions (scanning step of 0.06 eV). The binding energy axis of the analyzer was calibrated to the Au 4f_7/2_ (84.0 eV) region of the gold-covered sample placed at the same sample stage [52]. To avoid possible charging-related distortions, the binding energy scale was additionally controlled through the application of the procedure proposed by Greczynski and Hultman [53]. Obtained spectra underwent processing using CASA XPS^®^ software (version 2.3.25) with built-in algorithms (www.casaxps.com, accessed on 7 August 2024). If not specified, the particular components were fitted with a product of Gauss (30%) and Lorenz (70%) functions. Shirley’s function was applied for the background subtraction.

### 5.2. Scanning Electron Microscope Imaging

The morphology of prepared photocatalysts was analyzed using a field-emission scanning electron microscope (FE-SEM—FEI NovaNano SEM 450, FEI, Hillsboro, OR, USA) equipped with a Schottky gun. Images were obtained with the use of the Everhart–Thornley detector (ETD) or the through-lens detector (TLD) in immersion mode. The analysis of surface coverage by TNRs and AgNPs was estimated using Image J 1.52a software [54].

### 5.3. Band Gap Determination—Diffuse Reflectance Measurements

Diffuse reflectance (DRS) spectra were recorded using a UV-Vis spectrophotometer (USB2000+, Ocean Optics) equipped with a reflection/backscatter fiber probe in the spectral range of 200–800 nm. Barium sulfate (BaSO_4_, Sigma-Aldrich) was used as a reference. Then, the measured spectra were transformed using the Kubelka–Munk function, while the band gap energies of all samples were determined using the Tauc method described by Makuła et al. [55]. 

### 5.4. Photocatalytic Measurements

The photocatalysts obtained were immersed in PMMA cuvettes filled with 2.5 mL of rhodamine B (RhB) water solution with a concentration of 1 × 10^−5^ M. Before irradiation, cuvettes were placed in a dark room for 30 min, receiving an adsorption–desorption equilibrium. During the experiment, cuvettes with air access were placed in front of a xenon lamp (150 W, Instytut Fotonowy, Kraków, Poland) equipped with a UV or Vis cut-off filter at 15 cm (for UV) and 25 cm (for visible). By changing these distances, the intensity of radiation was the same for the UV and Vis ranges and equal to 223 mW/cm^2^. Changes in the RhB spectrum and concentration were monitored using a UV-Vis spectrophotometer (USB2000+, Ocean Optics, Dunedin, FL, USA).

## 6. Conclusions

The objective of this study was to synthesize various TiO_2_ nanostructures on a TiO_2_ substrate in order to cultivate 1D TiO_2_ nanorods (dense nanorods, nanorods, needle-like nanorods, nanoballs, and nanoflowers) using the hydrothermal method. AgNPs were grown by photoreducing Ag^+^ ions. This investigation examined the influence of various growth factors on the morphological development of TiO_2_ nanorods. This study involved the synthesis of various TiO_2_ nanostructures by adjusting three factors: (i) the ratio of acid to water in the chemical composition, (ii) the concentration of the precursor used in the hydrothermal process, and (iii) the duration of the hydrothermal heating reaction. The results suggest that altering the parameters leads to variations in the morphological structure, such as thin nanorods, thick nanorods, nanoflowers, and nanoballs. The photocatalytic efficacy of the TNRs and Ag-coated TNRs was evaluated by quantifying the degradation of the organic dye rhodamine B (RhB) under both ultraviolet (UV) radiation and visible light. The UV-Vis spectroscopy method was used to analyze the alterations in the RhB spectrum during photocatalytic processes. The results unambiguously demonstrate that exposure to UV light leads to the decolorization of the RhB solution, while exposure to visible light triggers the conversion of RhB into rhodamine 110, indicating a successful photocatalytic transformation. The modification of nanoball-like structures with the active metal silver (TNRs 4 Ag) demonstrated a significant increase in photocatalytic efficiency for various chemical composition parameters in both ultraviolet (UV) and visible light. Regarding the precursor amount parameter, TNRs 2 Ag (nanorod structure) exhibits higher efficiency under UV light, whereas the TNRs 6 Ag sample demonstrates greater effectiveness as a visible-light photocatalyst. The results highlight the potential of TiO_2_-based nanocomposites, particularly TNRs modified with AgNPs, for advancing photocatalytic technologies for environmental remediation and energy conversion applications.

## Figures and Tables

**Table 1:** Energy-dispersive X-ray spectroscopy (EDX) analysis of various TiO_2_ nanostructures.

Samples	Weight%			Atomic%		
	Ti	O	Ag	Ti	O	Ag
TNRs 1	2.72	2.28	----	3.98	1.59	----
TNRs 1 Ag	32.84	42.70	24.45	19.14	74.53	6.33
TNRs 2	51.91	48.89	----	26.50	73.50	----
TNRs 2 Ag	50.10	42.95	6.97	27.26	70.74	1.70
TNRs 3	61.47	38.60	----	34.70	65.30	----
TNRs 3 Ag	57.3	31.61	11.06	36.55	60.32	3.13
TNRs 4	67.03	32.97	----	40.45	59.55	----
TNRs 4 Ag	77.51	9.07	13.41	70.06	24.55	5.38
TNRs 5	2.73	3.56	----	4.08	1.56	----
TNRs 5 Ag	34.62	40.26	25.12	21.76	71.76	6.48
TNRs 6	62.12	37.88	----	35.39	64.61	----
TNRs 6 Ag	60.80	35.88	3.31	35.83	63.30	0.87
TNRs 7	46.33	53.67	----	22.38	77.62	----
TNRs 7 Ag	32.76	43.16	24.08	18.98	74.83	6.19
TNRs 8	62.33	37.67	----	35.59	64.41	----
TNRs 8 Ag	59.71	35.25	5.04	35.65	63.01	1.34

**Table 2:** Average length and diameter of TNRs family photocatalysts.

Type of Nanostructure	Thickness (from Cross-Section) [nm]	Length [nm]	Diameter [nm]	Diameter of Ag Particles [nm]
TNRs 1	-------	83 ± 20	13 ± 5	48 ± 23
TNRs 2	827 ± 34	863 ± 50	97 ± 22	34 ± 14
TNRs 3	612 ± 14	634 ± 57	285 ± 44	35 ± 20
TNRs 4	19,043 ± 72	2064 ± 142	4279 ± 416	50 ± 36
TNRs 5	226 ± 65	236 ± 33	23 ± 9	26 ± 17
TNRs 6	920 ± 50	929 ± 74	130 ± 18	33 ± 20
TNRs 7	103 ± 27	117 ± 30	20 ± 6	16 ± 11
TNRs 8	1056 ± 22	1133 ± 124	104 ± 21	24 ± 13

**Table 3:** The effect of synthesis parameters on the band gap E_g_ values.

Acid:Water Ratio		Amount of Precursor		Time of Reaction	
Material	E_g_ [eV]	Material	E_g_ [eV]	Material	E_g_ [eV]
TNRs 1	3.34	TNRs 5	3.38	TNRs 7	3.34
TNRs 1 Ag	3.30	TNRs 5 Ag	3.30	TNRs 7 Ag	3.30
TNRs 2	3.15	TNRs 2	3.15	TNRs 2	3.15
TNRs 2 Ag	3.08	TNRs 2 Ag	3.08	TNRs 2 Ag	3.08
TNRs 3	3.01	TNRs 6	3.07	TNRs 8	3.11
TNRs 3 Ag	1.98	TNRs 6 Ag	3.06	TNRs 8 Ag	3.08
TNRs 4	2.96	-	-	-	-
TNRs 4 Ag	2.99	-	-	-	-

**Table 4:** Reaction rate constants of TNRs and TNRs Ag for UV and Vis light and calculation of conversion efficiency for visible irradiation for RhB transformation.

Sample	UV	Visible		
	k [min^−1^]	k′ [min^−1^]	W_c_%	W_r_
TNRs 1	0.016	0.004	68	32
TNRs 1 Ag	0.030	0.005	60	40
TNRs 2	0.020	0.008	61	39
TNRs 2 Ag	0.029	0.009	50	50
TNRs 3	0.008	0.004	70	30
TNRs 3 Ag	0.021	0.007	67	33
TNRs 4	0.014	0.005	65	35
TNRs 4 Ag	0.033	0.015	50	50
TNRs 5	0.019	0.001	93	07
TNRs 5 Ag	0.029	0.004	78	22
TNRs 6	0.012	0.007	60	40
TNRs 6 Ag	0.022	0.009	48	52
TNRs 7	0.015	0.007	67	33
TNRs 7 Ag	0.009	0.006	65	35
TNRs 8	0.012	0.005	46	54
TNRs 8 Ag	0.013	0.005	46	54

**Table 5:** Various combinations of synthesis parameters.

Samples	HCl:H_2_O (mL)	TTBO (mL)	Time (hours)	Morphology
TNRs 1	50:40	1.5	4	Needle-like nanorods
TNRs 2	45:45	1.5	4	Nanorods
TNRs 3	25:65	1.5	4	Nanoflowers
TNRs 4	20:70	1.5	4	Nanoballs
TNRs 5	45:45	1	4	Needle-like nanorods
TNRs 6	45:45	2	4	Dense nanorods
TNRs 7	45:45	1.5	2	Needle-like nanorods
TNRs 8	45:45	1.5	6	Dense nanorods

## Data Availability

The original contributions presented in the study are included in the article. Further inquiries can be directed to the corresponding author.
